# Eighth Annual Report of the State Board of Health of Illinois

**Published:** 1887-02

**Authors:** 


### BOOK REVIEWS.

EIGHTH ANNUAL REPORT OF THE STATE BOARD OF HEALTH OF ILLINOIS, WITH AN APPENDIX. *pp. cxx, and 556.*

This is one of the most important reports of its kind ever issued in this country, and is itself sufficient evidence that the total expenditures of the Board for the year, viz: $12,831.26, were well invested.

Under the heading, "Medical Education," the Board reports very satisfactory progress.

There is not only a decided improvement in the standard and methods of education among the medical colleges of the United States, but there is also a gradual diminution in the number of students in attendance. The number of medical students attending the sessions of 1881–2, was 13,088, those of 1884–5 was 12,002, a loss of eight per centum. The number of medical graduates in 1881–2 was 4,500, and in 1884–5, 3,831, nearly sixteen per centum less. The Board attributes this condition of medical affairs to three causes, viz:

First, "hard times" and an increase in the number of students in attendance upon the Canadian schools. Second, a general and increasing desire on the part of the profession to elevate the standard of attainments necessary to enter its ranks. Third, the enforcement of certain requirements in states which have enacted laws regulating the practice of medicine. In this connection, the following facts taken from the reports of the per-centage of graduates to matriculates, at the colleges of the United States and Canada, is interesting: In Canada, the lowest is 6, the highest is 23. In the United

States the lowest is 12, the highest is 58.8, and nine of the colleges have a per-centage above 40. Among the states, Indiana, with a total of six colleges, has an average per-centage of 41.33, which is decidedly the worst showing. A comparison of the requirements for admission and graduation, shows the per-centage of graduates to matriculates highest in those colleges where the standard is lowest.

The contents of the appendix embraces: A, Report on Disinfection and Disinfectants; B, Coast Defenses Against Asiatic Cholera; C, Report of Proceedings, Sanitary Council of the Mississipi Valley; D, Meteorological Tables; E, State Sanitary Survey and House-to-house Inspection; F, Vital Statistics of Illinois, and Coroner's Inquest; G, Medical Education in the United States and Canada, 1765–1886. Of these *A* is the report of the Committee on Disinfectants appointed at the twelfth annual meeting of the American Public Health Association. It is very complete and of great practical value, but cannot be summarized in this notice. *G* contains an enumeration of all of the medical schools which exist or have existed in the countries mentioned, together with a statement, made out by the authorities of the institution reported, of the requirements for admission and graduation, and of their teaching force and facitities for instruction.

The titles of the other articles in the appendix explain themselves. A limited number of copies of this, and also of the seventh annual report, are still in possesion of the Board and may be obtained by physicians who apply for them through their senators or representatives. E. W.

